# Disrupted ADP-ribose metabolism with nuclear Poly (ADP-ribose) accumulation leads to different cell death pathways in presence of hydrogen peroxide in procyclic *Trypanosoma brucei*

**DOI:** 10.1186/s13071-016-1461-1

**Published:** 2016-03-23

**Authors:** Mariana Schlesinger, Salomé C. Vilchez Larrea, Teemu Haikarainen, Mohit Narwal, Harikanth Venkannagari, Mirtha M. Flawiá, Lari Lehtiö, Silvia H. Fernández Villamil

**Affiliations:** Instituto de Investigaciones en Ingeniería Genética y Biología Molecular “Dr. Héctor N. Torres”, Consejo Nacional de Investigaciones Científicas y Técnicas, Vuelta de Obligado 2490, 1428 Ciudad Autónoma de Buenos Aires, Argentina; Faculty of Biochemistry and Molecular Medicine & Biocenter Oulu, University of Oulu, P.O. Box 3000, FIN-90014 Oulu, Finland; Departamento de Química Biológica, Facultad de Farmacia y Bioquímica, Universidad de Buenos Aires, 1428 Ciudad Autónoma de Buenos Aires, Argentina

**Keywords:** PARP, PARG, PAR, *Trypanosoma brucei*, Genotoxic damage, Cell death

## Abstract

**Background:**

Poly(ADP-ribose) (PAR) metabolism participates in several biological processes such as DNA damage signaling and repair, which is a thoroughly studied function. PAR is synthesized by Poly(ADP-ribose) polymerase (PARP) and hydrolyzed by Poly(ADP-ribose) glycohydrolase (PARG). In contrast to human and other higher eukaryotes, *Trypanosoma brucei* contains only one PARP and PARG. Up to date, the function of these enzymes has remained elusive in this parasite. The aim of this work is to unravel the role that PAR plays in genotoxic stress response.

**Methods:**

The optimal conditions for the activity of purified recombinant *Tb*PARP were determined by using a fluorometric activity assay followed by screening of PARP inhibitors. Sensitivity to a genotoxic agent, H_2_O_2,_ was assessed by counting motile parasites over the total number in a Neubauer chamber, in presence of a potent PARP inhibitor as well as in procyclic transgenic lines which either down-regulate PARP or PARG, or over-express PARP. Triplicates were carried out for each condition tested and data significance was assessed with two-way Anova followed by Bonferroni test. Finally, PAR influence was studied in cell death pathways by flow cytometry.

**Results:**

Abolition of a functional PARP either by using potent inhibitors present or in PARP-silenced parasites had no effect on parasite growth in culture; however, PARP-inhibited and PARP down-regulated parasites presented an increased resistance against H_2_O_2_ treatment when compared to their wild type counterparts_._ PARP over-expressing and PARG-silenced parasites displayed polymer accumulation in the nucleus and, as expected, showed diminished resistance when exposed to the same genotoxic stimulus. Indeed, they suffered a necrotic death pathway, while an apoptosis-like mechanism was observed in control cultures. Surprisingly, PARP migrated to the nucleus and synthesized PAR only after a genomic stress in wild type parasites while PARG occurred always in this organelle.

**Conclusions:**

PARP over-expressing and PARG-silenced cells presented PAR accumulation in the nucleus, even in absence of oxidative stress. Procyclic death pathway after genotoxic damage depends on basal nuclear PAR. This evidence demonstrates that the polymer may have a toxic action by itself since the consequences of an exacerbated PARP activity cannot fully explain the increment in sensitivity observed here. Moreover, the unusual localization of PARP and PARG would reveal a novel regulatory mechanism, making them invaluable model systems.

**Electronic supplementary material:**

The online version of this article (doi:10.1186/s13071-016-1461-1) contains supplementary material, which is available to authorized users.

## Background

*Trypanosoma brucei* is the etiological agent of the sleeping sickness in humans and Nagana in cattle, in the region of Sub-Saharan Africa. According to the World Health Organization, African trypanosomosis is endemic in 24 African countries, with approximately 30,000 cases in 2010 (WHO, 2010). Current treatments depend on the stage of the affliction and the disease is typically diagnosed only after it has already advanced.

Poly(ADP-ribose)polymerases (PARPs) catalyze the formation of ADP-ribose polymer chains (PAR) by transferring the ADP-ribose region from NAD^+^ to certain residues in target proteins or to a nascent chain (PAR). Most of these enzymes also typically carry out an auto-modification reaction. The superfamily of human PARP comprises 17 proteins [1]. Among the functions they carry out, the participation of human PARP-1 (*h*PARP-1) in signalling and repair of harmed DNA has been the center of most of the research carried out in the field [2–8].

Poly(ADP-ribose) glycohydrolase (PARG) hydrolyzes the glycosidic bonds present in the polymer synthesized by PARP enzymes, re-establishing basal levels of PAR [3, 4, 9]. One PARG gene is encoded in the human genome and is expressed as several isoforms which occur in different cellular organelles [10]. It has been shown that the depletion of all such isoforms is lethal in embryonic mice stages [11]. On the other hand, cell-based models evidenced that the lack of PARG isoforms has no consequences on cell viability. However, PARG-deficient cells failed to amend strand breaks after genomic harm augmenting cell death; an outcome that points out the importance of PARG involvement in DNA damage response [12–14]. Illuzi *et al*. [15] highlighted that PARG tightly regulates the polymer levels produced upon genomic harm, avoiding injurious cell consequences caused by PAR over-accumulation. *Trypanosoma brucei* has only one PARG of 531 amino acids, sharing high sequence identity and similarity with the orthologous sequence in *Trypanosoma cruzi* [16]. To date, no functional studies for PARG in trypanosomatids have been reported.

Our earlier studies on PAR metabolism in trypanosomatids showed that the inhibition of *Tc*PARP negatively impacts on *T. cruzi* growth [17]. This result encouraged us to analyze the only PARP protein identified in *T. brucei* (*Tb*PARP). Here we describe the activity requirements for the enzyme and identify potent *Tb*PARP inhibitors. The most potent compounds were tested in *T. brucei* cultures to assess their effect on parasite growth and their ability to inhibit the polymer synthesis after a genotoxic stimulus. We have also tested the sensitivity of PARP over-expressing parasites, as well as PARP or PARG silenced parasites in oxidative stress conditions. Furthermore, we determined the cell death pathways involved in every case.

## Methods

### Protein expression

*Tb*PARP was cloned into a pET-22b + expression vector [18]. For expression, the pET-22b + vector bearing the *Tb*PARP gene was transformed to *E. coli* Rosetta2 (DE3) strain. Cells were grown in baffled flasks containing 750 mL of Terrific Broth (TB) auto-induction media with antibiotics (50 μg/mL ampicillin and 34 μg/mL chloramphenicol), glycerol 8 g/L and trace elements. Cell pellet was stored at -20 °C in lysis buffer (0.1 M HEPES pH 7.5, 500 mM Sodium Chloride, 10 % glycerol, 0.01 M imidazole, 500 μM TCEP, 0.05 % IGEPAL).

### Protein purification

Lysozyme 0.25 mg, benzonase 250 U (both compounds from Sigma-Aldrich), a protease inhibitor tablet (Roche), and 3-AB 1 mM (Alexis Biochemicals) were added to the thawed cells and samples were sonicated with 50 % duty cycle for 30 min (BRANSON 250 Sonifier). After centrifugation, the supernatant was filtered through a 0.45 μm syringe filter. Samples were loaded in HisTrap HP column (GE Healthcare) and washed with 10 mL of binding buffer (20 mM HEPES pH 7.5, 0.5 M NaCl, 10 mM imidazole, 10 % glycerol, 500 μM TCEP) using a peristaltic pump at 4 °C. The column was washed at room temperature with 25 mM imidazole binding buffer and eluted with 250 mM imidazole binding buffer. Elute was divided into four and each sample was further purified by size exclusion chromatography using a Superdex 200 High Load 10/30 column (GE Healthcare) with binding buffer. Fractions with higher activity were pooled and flash frozen as small aliquots to be stored at -70 °C.

### Activity assay optimization

The optimal conditions for the activity of purified recombinant *Tb*PARP, were determined by using a fluorometric activity assay [19]. Incubation at 25 °C with shaking at 300 rpm was placed in propylene plates of 96 wells (Greiner BioOne). To terminate the reaction 2 M KOH (20 μL) and 20 % acetophenone in ethanol (20 μL) were added to each well. After incubating for 10 min at 4 °C, 90 μl of 88 % formic acid was added and incubated in an oven at 110 °C for 5 min. This reaction converts remaining NAD^+^ to a fluorescent derivative. After incubation, the plate was allowed to cool down for 15 min and fluorescence was measured using excitation wavelengths of 372 nm and 444 nm for excitation and emission, respectively (Varioskan Flash 4.00.53, Thermo Scientific). Consumption of NAD^+^ was detected by the decrease in fluorescence. Experiments were done in triplicates. The activity in control wells were kept under 25 % by optimizing the protein concentration and the incubation time, in order to get a robust signal without slowing down the enzyme activity.

Different assay buffers were tested (Additional file 1) and the buffering agent was found to have a large impact on enzymatic activity. The optimal activity was obtained with phosphate (Na) or phosphate (K) buffer at pH 7. Importantly, *Tb*PARP activity increases as a function of nicked DNA (activated DNA) concentration. Maximum *Tb*PARP activation was achieved at 25 μg/mL (Additional file 1) causing an 18-fold increase in *Tb*PARP activity. A small set of cations was also tested in order to verify whether the enzymatic activity would depend on them. Most divalent cations resulted in decreased *Tb*PARP activity, with Ni^2+^, Mn^2+^,Ca^2+^, and Zn^2+^ showing the greatest inhibition, while Mg^2+,^ a known co-factor of enzymes that participate in DNA metabolism, increased *Tb*PARP activity in vitro 2-fold (Additional file 1). As stabilizing agents were tested, we observed that the addition of BSA was beneficial to maintain *Tb*PARP activity when enzyme concentration was in nM range. Notably, the addition of both BSA and Mg^2+^ showed no increase in *Tb*PARP activity when compared to the addition of BSA alone and therefore Mg^2+^ was not added in the reaction buffer for further experiments. The final assay buffer was 100 mM Na_2_HPO_4_/NaH_2_PO_4_ pH 7, 500 μM TCEP, 1 μg/μL BSA, and 0.025 mg/mL activated DNA (Sigma Aldrich) and 500 nM NAD^+^ (Sigma Aldrich).

### Screening of *Tb*PARP inhibitors

Thirty - one compounds were tested. Dilutions from DMSO stocks were made (100 μM, 10 μM and 1 μM) and aliquots of those were used in the enzymatic reaction with 10-fold dilution. Inhibitor solutions were added in triplicates to the plate, followed by the addition of the NAD^+^. Reactions were initiated by addition of *Tb*PARP diluted in the same buffer (10 nM). The plate was incubated at 25 °C with shaking (300 rpm, Biosan PST-100 HL) for 45 min. This resulted in consumption of 50 % of the NAD^+^ and signal to background ratio of 2. To detect any potential fluorescence inherent in the compounds, control reactions were carried out separately with compound alone at 10 μM final concentration. The possible fluorescence quenching properties were controlled using wells within the experimental plate containing the compound with 500 nM NAD^+^ in the assay buffer. Wells with only NAD^+^ present were considered as 100 % inhibition and wells with NAD^+^ and PARP present were considered as 0 % inhibition.

Homogenous assay results were confirmed for the best inhibitors using biotinylated NAD^+^ as a substrate (bioNAD+, Trevigen). Incubation mixture consisted of *Tb*PARP (120 nM), bioNAD^+^ (1 μM) and nicked DNA (25 μg/mL) (Sigma Aldrich) in 0.1 M sodium phosphate buffer (pH 7) at room temperature. The assay was performed in absence (positive control) or in presence of 1 μM inhibitors, after which SDS sample buffer was added to stop the reaction by heating at 98 °C for 5 min. *Tb*PARP (120 nM) was also added to the reaction immediately before stopping it (negative control). PAR-biotinylated proteins were identified with streptavidin-conjugated horseradish peroxidase (PerkinElmer) after each reaction mix was run on SDS-PAGE and transferred onto a membrane of nitrocellulose.

### Potency of inhibitors

The half maximal inhibitory concentration (IC_50_) was obtained for the most potent *Tb*PARP inhibitors: Veliparib (ABT-888, Alexis Biochemicals), 4-ANI (Alexis Biochemicals), EB-47 (Alexis Biochemicals), 1,5-Isoquinolinediol (Alexis Biochemicals), NU1025 (Alexis Biochemicals), PJ-34 (Alexis Biochemicals), Olaparib (JS Research Chemicals Trading), RF03876 (Maybridge), and Rucaparib (Selleck Biochemicals). The IC_50_ value for 3-AB (Alexis Biochemicals) was also determined to be used as a reference. Briefly, reaction mix together with the inhibitors at half log dilutions were added to a 96-well plate (10 data points). Reactions were incubated for 11 min at 25 °C after initiating the reaction with the addition of 5 nM *Tb*PARP at a final volume of 50 μL. Measurement of the remaining NAD^+^ is described in section *Screening of TbPARP inhibitors*. All reactions were performed in triplicates and three independent dose response curves were fitted for each inhibitor. Control wells with only NAD^+^ or NAD^+^ and enzyme were used in the curve fitting, as detailed in our previous work [17].

### Structural analysis

*Tb*PARP homologues [UniProt:Q0PW89] were searched from Protein Data Bank. *h*PARP-1 [PDB: 3GJW] [20], *h*PARP-2 [PDB:3KJD] [21] and *h*PARP-3 [PDB:3C4H] [22] crystal structures were chosen based on homology and the quality of the structures, and aligned to *Tb*PARP with ClustalW [23]. Aline [24] was used for the analysis of the sequence alignment. *Tb*PARP regulatory and catalytic domains were modelled with Modeller [25], based on *h*PARP-1 structure. SSM superposition algorithm implemented in COOT [26, 27] was utilized for structural superpositions. Structural figures were made with CCP4mg [28].

### Parasite cultures and *in culture* inhibition of *Tb*PARP

*T. brucei* procyclic strain 29–13 [29] was cultured at 28 °C in SDM-79 (Bioscience) containing 10 % (v/v) FCS and 0002 % hemin. *T. brucei* bloodstream strain 427 90-13 [29] was cultured at 37 °C in HMI-11 (Iscore’s Modified Dulbecco’s Medium (Invitrogen), 100 mg/L sodium pyruvate, 136.1 mg/L hypoxantine, 38.7 mg/L thymidine, 28.22 mg/L bathocuproinedisulfonic acid, 181.8 mg/L L-cysteine, 3.024 mg/L sodium carbonate, 196 μM β-mercaptoethanol) containing FCS (10 % v/v). Parasite viability was analyzed by microscopy.

For *in culture* inhibition assays, parasites of the procyclic form of *Trypanosoma brucei* were grown for 48 h up to a density of 5×10^6^ parasites/mL. Parasites of the bloodstream form were grown for 24 h up to a density of 5×10^5^ parasites/mL. In both cases, cells were harvested and preincubated for 30 min or 10 min in PBS-Glucose 2 % with inhibitors added, after which the parasites were treated with 500 μM or 250 μM hydrogen peroxide for procyclic and bloodstream forms, respectively, for 10 min. Protein extracts were prepared as indicated in our previous work [17], and 3 μg of total protein were manually spotted onto a membrane of nitrocellulose (GE Healthcare) for Dot blot analysis revealed with by commercial PAR antibody (BD).

### Effect of the inhibitors on parasite growth

*Trypanosoma brucei* procyclic parasites were grown in SDM-79 medium for 48 h until reaching a density of 5×10^6^ cells/mL. Aliquots of 200 μl were distributed in 96-well plates and inhibitors were present at different final concentrations, as indicated in the figures. Nifurtimox (NFX) was used as a positive control in concentrations ranging from 0.25–25 μM. Culture density was checked by OD_600_ after 48 h.

*Trypanosoma brucei* bloodstream parasites were grown in HMI-11 medium for 24 h until reaching a density of 5×10^5^ cells/mL. Aliquots of 100 μL were distributed in 96-well plates and inhibitors were added at different concentrations as indicated in the figures. After an incubation period of 24 h, the parasite number was counted with a Neubauer chamber.

In all experiments, triplicates were done for each condition tested and data significance was assessed by one-way Anova (GraphPad Prism5.03 version Software).

### PARP and PARG down-regulated *T. brucei* lines and PARP over-expressing *T. brucei* line

A fragment of the *Tb*PARP cDNA (nucleotides 1030 to 1445) [GenBank:DQ679800, Tb927.5.3050] and a fragment of the *Tb*PARG cDNA (nucleotides 593 to 979) [Tb927.9.12810] were sub-cloned into the tetracycline-inducible expression vector p2T7^TI^-177 [30] to down-regulate the genes of interest. In addition, whole *Tb*PARP cDNA sequence was sub-cloned into the expression vector p2216 [31] (PCR primer sequences available upon request) to over-express the PARP-eYFP fusion gene.

Not I-linearized RNAi-*Tb*PARP and RNAi-*Tb*PARG constructs and the over-expression construct (10 μg) were transfected into procyclic parasites by electroporation, as described by Downey *et al*. [32]. The selection of the transfectants was carried out with 20 μg/mL zeocin, and dsRNA and over-expression of the fusion protein was induced with 1.0 μg/mL tetracycline for 3 days.

Gene expression knock- down was confirmed by Northern-blot and Western-blot analysis and gene over-expression was confirmed by Western blot with specific antibodies.

### Effect of hydrogen peroxide (H_2_O_2_) in cell survival

Cell survival was determined by incubating 500 μL of 1 × 10^6^ parasites/mL culture in 24-well cell culture plates (Cellstar) for 6 h with different concentrations of H_2_O_2_ (30 μM, 95 μM, 300 μM and 950 μM) and counting motile parasites over the total number using a Neubauer chamber. Each condition was tested in triplicates. Statistical significance was assessed with two-way Anova followed by Bonferroni test (version 5.03 of GraphPad Prism for Windows).

Wild type procyclic culture was preincubated for 30 min with Olaparib at 29 nM concentration in order to measure cell survival, compared to the culture with no inhibitor added (control). The addition of DMSO in the same concentration as it is present in Olaparib experiment did not show any difference compared to the control culture.

Transgenic RNAi-*Tb*PARP (p2T7-*Tb*PARP), RNAi-*Tb*PARG (p2T7-*Tb*PARG) and *Tb*PARP over-expressing (p2216-*Tb*PARP) procyclic cultures induced for 3 days with tetracycline (1 μg/mL) were also challenged with H_2_O_2_ and compared to the non-induced cultures.

### Analysis of PAR formation after hydrogen peroxide (H_2_O_2_) treatment by Western blot

Parasites were treated for 10 min with 1 mM H_2_O_2_. Western blot analysis was carried out as it is detailed in our previous work [17] with 1:5.000 polyclonal anti-PAR antibody (BD) made in rabbit. As a loading control we used α-tubulin antibody.

### Immunolocalization of poly(ADP-ribose)

Immunolocalization experiments were carried out as detailed in our previous work [33] with 1:500 polyclonal anti- PAR antibody (BD) made in rabbit to detect PAR and 1:100 polyclonal anti-*Tb*PARP antibody (GenScript) made in rabbit to detect *Tb*PARP. Nuclear and kinetoplast DNA were stained with DAPI (2 μg/mL) (Sigma).

### Nucleus Fluorescence Measurement

Nucleus fluorescence measurement was obtained following ImageJ instructions. The area of interest was selected and parameters such as integrated density, area and mean grey value were measured by the software. Three different regions next to the nucleus were also selected as the background. Finally, corrected nucleus fluorescence was calculated with the formula: CTCF = Integrated Density - (Area of selected nucleus × Mean fluorescence of background readings).

### Annexin V flow cytometric analysis

Parasites were treated with 1 mM H_2_O_2_ for different time intervals and the cell death pathway involved was assessed as it has been detailed in our previous work [34]. The exposure of wild type procyclic parasites for 6 and 12 h to 10 μg/mL Concanavalin A type IV (Sigma-Aldrich) confirmed the apoptosis-like death pathway (positive control) [35, 36]. Transgenic *Tb*PARP over-expressing (p2216-*Tb*PARP) and RNAi-*Tb*PARG (p2T7-*Tb*PARG) procyclic cultures were previously induced for 3 days with tetracycline (1 μg/mL). Results were analyzed with Cyflogic v 1.2.1 software.

## Results

### *In vitro* screening of compounds that inhibit *Tb*PARP

**Fig. 1:**
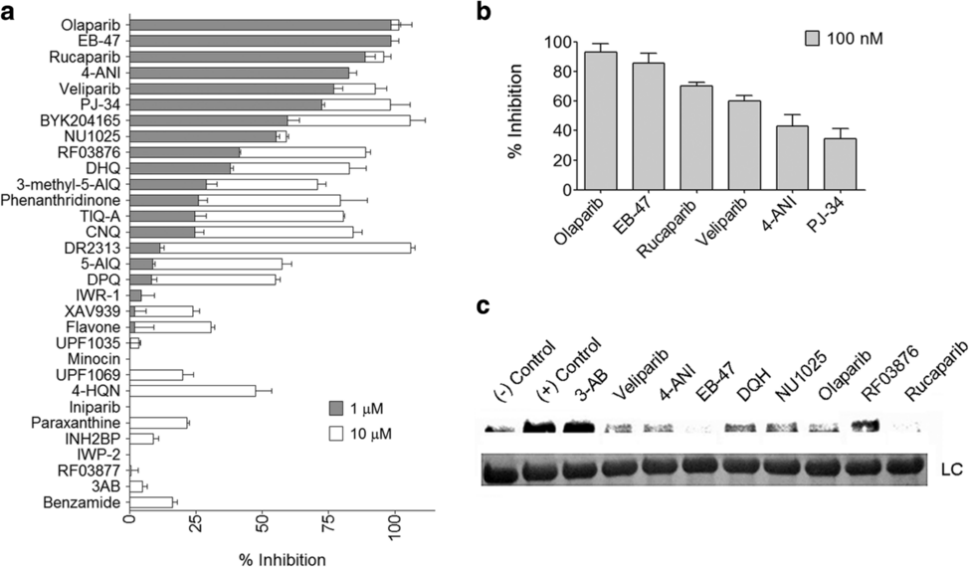
Screening of *Tb*PARP inhibitors. **a** Inhibition of the *in vitro* *Tb*PARP (2.5 nM) activity was tested with a library of 31 compounds. Inhibitors were added at 1 μM (grey bars) or 10 μM (white bars). The values were transformed to % of inhibition. Triplicates were measured for every data point, and the mean and the standard deviation are shown. **b** For the most potent inhibitors, % inhibition was determined at 100 nM concentration. **c** Inhibition of *Tb*PARP was confirmed in the presence of 1 μM inhibitors by Western blot using 1 μM biotinylated NAD^+^ as a substrate and in the presence of activated DNA 25 μg/mL. *Tb*PARP (120 nM) was incubated in the absence of inhibitors for one hour (positive control) or was added immediately before stopping the reaction (negative control). Synthesized biotinylated PAR was recognized with streptavidin-HRP. Loading of equal amount of protein in every lane was checked with Lysozyme

Thirty one compounds, most of them previously reported to inhibit PARP enzymes, were tested to assess their ability to abrogate *Tb*PARP activity *in vitro* (Additional file 2: Table S1) [17]. The compounds were analyzed by the described activity assay at concentrations of 1 and 10 μM (Fig. 1a). DMSO concentration was kept under 1 % in all experiments described below. Compounds that demonstrated to have apparent IC_50_ values higher than 1 μM were retested at a lower concentration (100 nM) (Fig. 1b) and the results were corroborated by Western Blot, in which inhibitors were added to the reaction at 1 μM concentration together with the biotinylated NAD^+^ substrate (bioNAD^+^) (Fig. 1c). Afterwards, IC_50_ values were experimentally measured for the best inhibitors (Table 1). The compounds with the most potent inhibiting ability were Olaparib (IC_50_ 2.9 nM), EB -47 (IC_50_ 6.5 nM), 4-ANI (IC_50_ 23 nM), and Veliparib (IC_50_ 49 nM) (Table 1). These same inhibitors had previously been demonstrated to be the most potent inhibitors for the orthologous enzyme in *T. cruzi* [17].

**Table 1:** The most potent inhibitors of TbPARP

Compound	TbPARP	TbPARP	hPARP-1	Reference
	IC_50_/μM	pIC_50_ ± SEM	IC_50_ (K_i_)/μM	
3-AB	58	4.24 ± 0.192	22	[50]
Veliparib	0.049	7.31 ± 0.073	0.005 (K_i_)	[51]
4-ANI	0.023	7.64 ± 0.198	0.18	[50]
EB-47	0.0065	8.19 ± 0.184	0.045	[52]
1,5-Isoquinolinediol	1.2	5.91 ± 0.072	0.39	[50]
NU1025	0.68	6.17 ± 0.046	0.4	[53]
PJ-34	0.58	6.23 ± 0.109	0.02	[52]
Olaparib	0.0029	8.53 ± 0.104	0.005	[54]
RF03876	1.1	5.94 ± 0.166	-	-
Rucaparib	0.15	6.83 ± 0.032	0.0014 (K_i_)	[55]

Potency values are compared with those reported for human PARP-1. Potency values are means of the three dose-response curves fitted separately

### Homology modeling and inhibitor selectivity

**Fig. 2:**
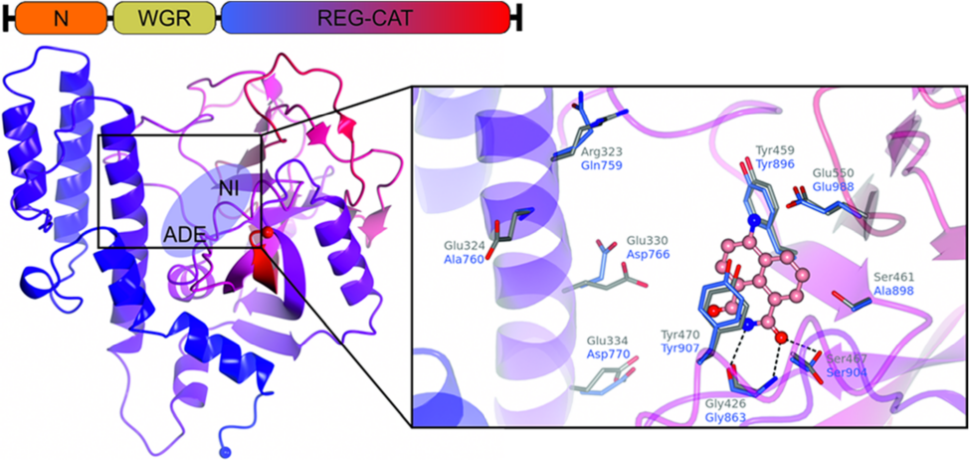
Template-based model. Domain organization and homology model of the REG-CAT region of *Tb*PARP, with nicotinamide (NI) and adenosine (ADE) binding pockets of the substrate NAD^+^ labelled. The inset shows how 4-ANI binds to the binding site of nicotinamide (built from the chicken PARP superposition PDB code 2PAX [56]). Residues around the NI site and at the REG domain are shown for *Tb*PARP (grey) and 2PAX (human PARP-1 numbering, blue)

Inhibitor binding to *Tb*PARP was evaluated by generating a homology model based on human PARP crystal structures. Sequence alignment included PARP regulatory (REG) and ADP-ribosyl transferase (CAT) domains as they are conserved in *h*PARP1-3 and in *Tb*PARP [17]. Importantly, the donor NAD^+^ binding site is highly conserved between these proteins. The nicotinamide subsite (NI) contains the common interactions utilized by many PARP inhibitors, as the inhibitors form a π-stacking interaction with Tyr907 and hydrogen bonds to Gly863 and to Ser904 (Fig. 2). This is demonstrated with the superposition of chicken PARP-1 in complex with 4-ANI (PDB accession code 2PAX) and *Tb*PARP homology model. However, there are a few differences between the REG domains of the *h*PARP-1 and *Tb*PARP that may affect the binding of larger inhibitors that extend out of the NI pocket, such as PJ-34 and Rucaparib, which show selectivity towards *h*PARP-1 (Fig. 2).

### *In culture* inhibition of PARP from *Trypanosoma brucei*

**Fig. 3:**
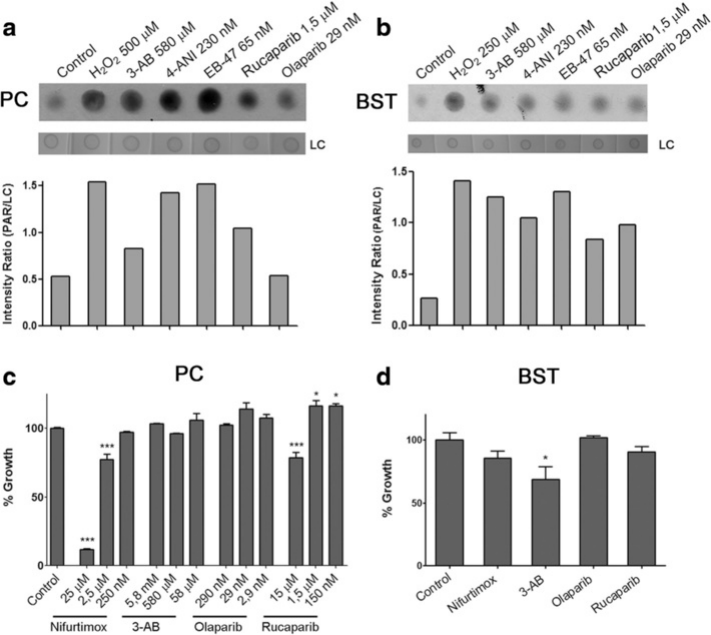
Effect of *in culture* inhibition of *Tb*PARP on parasite growth. PAR formation was assessed by Dot Blot (**a**) in procyclic cultures pre-incubated for 30 min with PARP inhibitors and treated with 500 μM H_2_O_2_ for 10 min, and (**b**) in bloodstream cultures pre-incubated for 10 min with PARP inhibitors and treated with 250 μM H_2_O_2_. Positive controls (H_2_O_2_) correspond to those reactions with no inhibitors added, while negative controls (control) correspond to parasites not treated with H_2_O_2_. The membranes were stained with Ponceau Red as a loading control (LC). Lower panels in both cases show the intensity ratio of PAR to LC signals calculated using ImageJ software. **c** Effect of the selected PARP inhibitors on procyclic parasites’ growth after 48 h incubation. **d** Effect of the selected PARP inhibitors on bloodstream parasites’ growth after 24 h incubation. Nifurtimox was used at 2.5 μM and inhibitors were used at the same concentrations as in panel B. Culture growth in absence of inhibitors or Nifurtimox was considered as 100 %. Triplicates were performed for every data point, and expressed as means and standard deviations. Statistical significance is specified in comparison to control groups (***, *P* < 0.001; **, *P* < 0.01; *, *P* < 0.05)

The compounds that were identified as the best inhibitors of *Tb*PARP *in vitro* were tested for their effectiveness to inhibit *in culture* PAR synthesis on both procyclic and bloodstream forms of *T. brucei.* The parasites were challenged with hydrogen peroxide, a DNA damaging agent [18, 34]. The inhibitors were tested *in culture* at a concentration 10-fold higher than the *in vitro* IC_50_ value. The compounds and concentrations were the following: 0.23 μM 4-ANI; 65 nM EB-47; 29 nM Olaparib and1.5 μM Rucaparib. 3-AB at 580 μM was also tested since it is a well-known PARP inhibitor. In the case of procyclic parasites, Olaparib and Rucaparib inhibited PAR formation *in culture* while 4-ANI scarcely inhibited the enzyme and EB-47 did not inhibit *Tb*PARP at all (Fig. 3a). Most of the inhibitors diminished, but didn’t inhibit completely PAR formation in the bloodstream stage of *T. brucei* at the indicated concentrations (Fig. 3b).

### PARP is not essential for *T. brucei* growth

Olaparib and Rucaparib, which demonstrated to inhibit PAR formation *in culture* more effectively than the rest of the compounds, were selected to be evaluated on their ability to affect procyclic parasite growth in a series of concentrations ranging from the *in vitro* IC_50_ value up to two orders of magnitude higher. 3-AB, the inhibitor reported to diminish *T. cruzi* growth, was also tested. Nifurtimox was also included in the panel of compounds as a positive control due to its extensively proven trypanocidal activity [37, 38]. None of the PARP inhibitors tested were able to negatively affect parasite growth *in culture* (Fig. 3c), except for Rucaparib, which caused a 20 % decrease in growth at the highest 15 μM concentration. Nifurtimox, in contrast, slowed down procyclic parasite growth already at a 2.5 μM concentration. Growth curves were measured for the bloodstream form parasites in the presence of Olaparib (29 nM) and Rucaparib (1.5 μM), and despite that they were able to inhibit PAR formations *in culture* they were not able to significantly affect *T. brucei* growth or survival (Fig. 3b). Incubation in the presence of 3-AB or Nifurtimox, however, did provoke a decrease on bloodstream parasite growth; although only the effect of 3-AB was significant when compared to the control (Fig. 3d).

As these results are in contrast with our previous results on *T. cruzi* [17], we set out to verify them by knocking down the enzyme of interest by interference RNA. The experiments performed with transgenic RNAi-*Tb*PARP procyclic parasites indeed indicate that the lack of *Tb*PARP does not alter the culture growing rate (Additional file 3). Since the protein was not completely silenced and the low PARP amount remaining could be enough for survival in regular growth conditions, we evaluated Tet induced RNAi-*Tb*PARP procyclic parasites in the presence of 290 nM Olaparib. A similar result was obtained, with no change in growth rate (Additional file 3). Overall, the experiments suggest that *Tb*PARP would not be indispensable for the procyclic form of *T. brucei* under standard conditions.

### Sensitivity of procyclic *Trypanosoma brucei* to oxidative stress

**Fig. 4:**
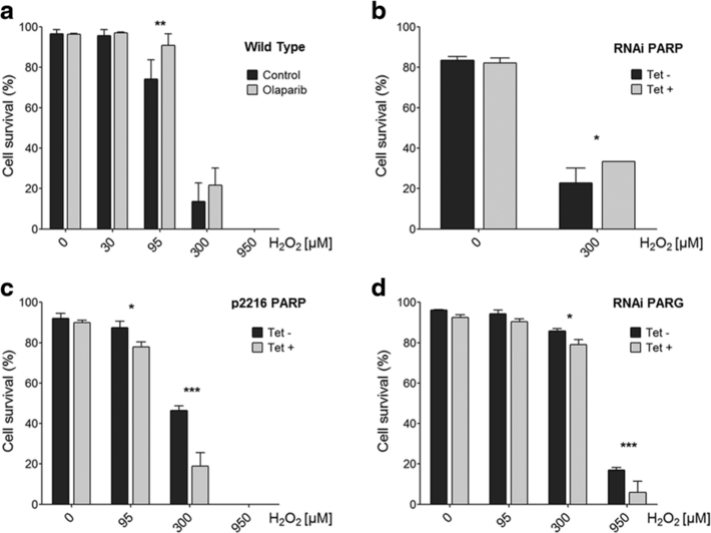
Cell survival in cultures with a modified PAR synthesis subjected to hydrogen peroxide (H_2_O_2_) treatment. Cell survival was assessed by measuring parasite motility 6 h after treatment with different H_2_O_2_ concentrations. **a** Procyclic cultures previously incubated with 29 nM Olaparib for 30 min and with no inhibitor added (control). **b** Three day-induced (Tet +) and non-induced (Tet -) transgenic RNAi-*Tb*PARP cultures, (**c**) Three day-induced (Tet +) and non-induced (Tet -) transgenic *Tb*PARP over- expressing cultures and (**d**) Three day-induced (Tet +) and non-induced (Tet -) transgenic RNAi-*Tb*PARG cultures. Three independent experiments were carried out for every case. Statistical significance is specified in comparison to control groups (***, *P* < 0.001; **, *P* < 0.01; *, *P* < 0.05)

Given that *Tb*PARP is not crucial for the survival of the procyclic form, we next tried to determine the possible role of *Tb*PARP in genomic harm signaling and repair processes. As a parameter of the sensitivity of *T. brucei* procyclic parasites to the oxidative stress, we determined the response to the genotoxic compound hydrogen peroxide (H_2_O_2_). We estimated the parasite survival by measuring motility 6 h after the treatment with this agent in parasites with no functional PARP. Olaparib was chosen due to its inhibitory potency towards *Tb*PARP (Table 1). When low doses of hydrogen peroxide (30 μM) were used, there was no difference in parasite viability in presence or absence of Olaparib at a concentration that has been proven to inhibit effectively PAR formation *in culture* (29 nM). However at 95 μM H_2_O_2_, a concentration that diminishes the number of motile parasites, the presence of Olaparib in the medium reduced the loss of viability induced by the oxidant and cultures became more resistant to this genotoxic agent (Fig. 4a). At a lethal H_2_O_2_ concentration (300 μM) there was a significant decrease in parasite motility both in absence or presence of Olaparib, however a slight protective effect could be observed in the presence of this compound, though not statistically significant. A similar result could be observed when we compared the effect of H_2_O_2_ on 3 day-induced transgenic RNAi-*Tb*PARP procyclic cultures to the non-induced ones subjected to the same treatment (Fig. 4b). Altogether, these experiments demonstrate that the lack of a functional *Tb*PARP leads to an increase in the resistance against hydrogen peroxide in procyclic *T. brucei* parasites.

PARGs are responsible for the break-down of PAR. We have identified only one protein that belongs to the PARG family in *T. brucei* (*Tb*PARG*)*, which expresses in both stages of the parasite (Additional file 4B). This enzyme has an expected molecular weight of 60 kDa. Moreover, it is always located in the nucleus in procyclic cultures, independently of the occurrence of genomic harm (Additional file 4A). This is similar to what we observed earlier for *T. cruzi* PARG (*Tc*PARG) [33].

In order to test the importance of PAR on the sensitivity towards oxidative stress we down- regulated PARG expression by RNAi and also obtained parasites over expressing *Tb*PARP (Additional file 5 A, B and C). PARG silencing or increased PARP expression did not cause morphological alterations but culture growth rate was slightly diminished in both cases (data not shown). Nevertheless, when we analyzed cell death by FACS, there was no difference in distribution of population between wild type, RNAi-*Tb*PARG or PARP over-expressing parasites in fresh cultures, as it will be shown in the next sections. In a similar experiment as the one described in Fig. 4a and b, we analyzed the effect of H_2_O_2_ on RNAi-*Tb*PARG and PARP over-expressing parasites, induced for 3 days. Both transgenic cells showed increased sensitivity to hydrogen peroxide treatment, as compared with the control (Fig. 4c, PARP over-expressing and 4D, PARG silenced parasites). In the case of the transgenic line over-expressing the PARP-eYFP fusion protein, we confirmed that this outcome is specific of an augmented expression of PARP, since transgenic eYFP over-expressing parasites presented a milder effect compared to the PARP over-expressing counterparts (Additional file 6).

### Nuclear PAR mediates apoptosis-like to necrosis switch in severe oxidative stress

**Fig. 5:**
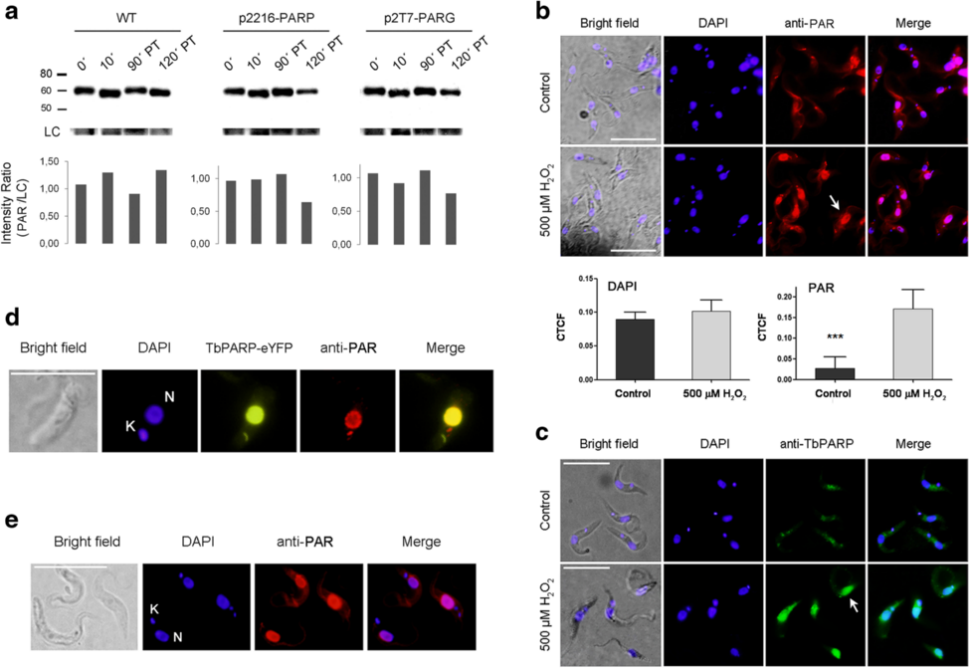
PAR response and localization in procyclic cultures subjected to hydrogen peroxide (H_2_O_2_) treatment. **a** Analysis by Western Blot of poly(ADP-ribose) formation revealed with anti-PAR antibody (BD) after 1 mM H_2_O_2_ treatment for 10 min in wild type (WT), PARP over-expressing (p2216-*Tb*PARP) and RNAi-*Tb*PARG (p2T7-*Tb*PARG) cultures. Data were normalized to anti-α tubulin band (LC) and are shown as the ratio of PAR to LC signals. Untreated (control) and procyclic cultures exposed to 500 μM H_2_O_2_ for 10 min were analyzed for PAR and PARP localization: (**b**) PAR was detected with specific polyclonal antibodies (BD) and nuclear PAR signal (arrow) quantification is shown below. The corrected total nucleus fluorescence (CTCF) was calculated as = Integrated Density - (Mean fluorescence of background readings X Area of selected nucleus). A Student Test was performed and significance of the nuclear signal in treated versus control parasites is indicated (*** *P* < 0.001). **c** *Tb*PARP localization was detected with specific polyclonal antibodies (GeneScript) in untreated (control) and procyclic cultures exposed to 500 μM H_2_O_2_ for 10 min (arrow). **d** *Tb*PARP-eYFP fusion protein localization was recognized by eYFP fluorescence and PAR localization was recognized with polyclonal anti-PAR antibody in a 3 day-induced *Tb*PARP over-expressing cultures (p2216-*Tb*PARP). **e** PAR localization was recognized with polyclonal anti-PAR antibody in a 3 day-induced RNAi-*Tb*PARG cultures (p2T7-*Tb*PARG). DAPI was used to identify nuclear (N) and kinetoplastid (K) DNA. White bar represents 10 μm

Based on these results we decided to examine the consequence of PAR metabolism alteration on H_2_O_2_-induced cell death. We treated parasites with 500 μM H_2_O_2_ and, as shown in Fig. 5a, the amount of PAR in wild type parasites increased after 10 min, diminished partially within the first 90 min and then increased again, probably due to DNA damage still present in the nucleus. When we examined the dynamics of PAR synthesis and degradation in PARP-over expressing or PARG-silenced procyclic forms of *T. brucei,* we observed a different pattern in the amount of PAR 90 and 120 min after a genotoxic stimulus, compared to wild type cultures (Fig. 5a). Intracellular PAR levels in wild type parasites are usually high (Fig. 5a 0 min) and located in the cytoplasm (Fig. 5b). After a genotoxic stimulus, an extensive quantity of PAR was synthesized in the nucleus and PARP translocated to this organelle (Fig. 5b and c). PAR signal in the nucleus was further quantified and it was shown that the difference between treated and control parasites was significant. Moreover, it is worth noting that both *Tb*PARG-silenced and *Tb*PARP-over expressing parasites showed PAR accumulation in the nucleus even in the absence of a genotoxic stimulus (Fig. 5d and e, and Additional file 7). *Tb*PARP-eYFP fusion protein was also recruited to the nucleus; supporting these results (Fig. 5d, Additional file 5).

**Fig. 6:**
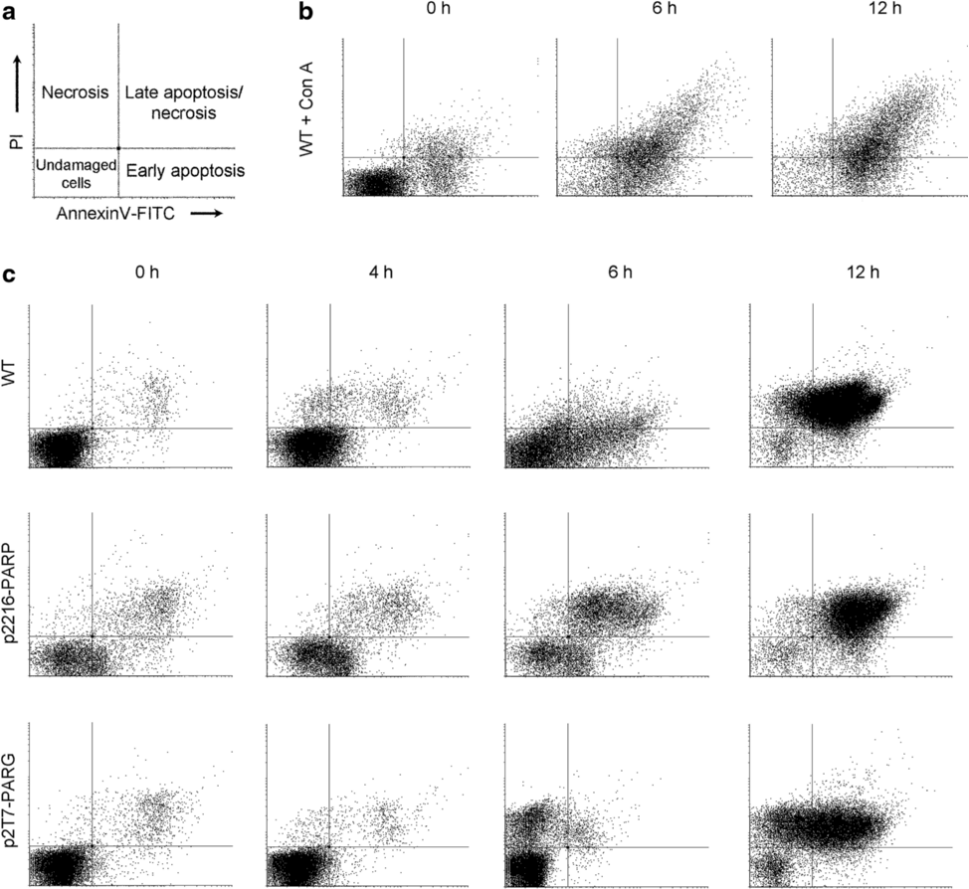
Cell death analysis by flow cytometry. **a** The diagram represents cell subpopulations identified by staining with propidium iodide (PI) and Annexin V-FITC conjugate. **b** Wild type culture was treated with 10 μg/mL of Concanavalin A (Con A) as an apoptosis-like control. **c** Diagram of wild type (WT), *Tb*PARP over-expressing (p2216-*Tb*PARP) and *Tb*PARG down-regulated (p2T7-*Tb*PARG) procyclic cultures after 1 mM H_2_O_2_ treatment for different time intervals. Transgenic parasites were previously induced for three days

We further investigate the mode in which hydrogen peroxide induces cell death in these parasites. Wild type and transgenic cultures were analyzed for phospholipid redistribution and PI incorporation by flow cytometry. Concanavalin A is well known to cause an apoptotic-like death in *T. brucei* [35, 36] and control cultures exposed to this lectin (10 μg/mL) showed the typical apoptosis response with an increment in Annexin incorporation (Fig. 6b). A similar pattern to apoptosis control was observed in wild type cells 6 h after H_2_O_2_ injury (Fig. 6c). However, when cells with modified PAR metabolism were treated, a pattern with an earlier incorporation of PI was obtained in both transgenic cultures, displaying a necrotic death signal. It should be taken into account that PARP-over expressing parasites display an apparent basal apoptosis-like pattern at time 0 h that is caused by the intrinsic eYFP fluorescence from the fusion protein; however, the overall process shows a necrotic-like death pathway (Fig. 6c).

## Discussion

Previously, we have demonstrated that *T. brucei* presents only one PARP protein (*Tb*PARP) [18]. Here, in an attempt to describe its enzymatic requirements, we proved *Tb*PARP is highly activated by damaged DNA, in agreement with our previous report for the trypanosomatids *C. fasciculata* [39] and *T. cruzi* [18], and this is also in-line with the behaviour described for *h*PARP-1 and 2 [1]. The N-terminus of *T. brucei* PARP is abundant in basic amino acids and probably the region responsible for DNA strand break-detection and activity modulation [18]. *Tb*PARP does not require any metal ions to carry out its activity. The divalent cations such as Mn^2+^, Ni^2+^or Zn^2+^ resulted in an inhibitory effect probably because these ions could bind to important sulphydryl groups.

The optimization of the reaction conditions allowed the use of an activity assay based on the fluorescence [17, 19] and helped us to identify specific inhibitors towards *Tb*PARP from a library of PARP inhibitors. Similarly to *Tc*PARP, the most potent *Tb*PARP inhibitors identified were Olaparib, EB-47, 4-ANI, Veliparib, and Rucaparib. Mostly, the similarities in the IC_50_ values among *Tb*PARP, *Tc*PARP, and *h*PARP-1 can be explained by the conservation of the donor NAD^+^ binding pocket, as evidenced by the homology model (Table 1, Fig. 2). Rucaparib binds to the nicotinamide binding pocket of PARPs [40] and lower potency of Rucaparib towards *Tb*PARP (150 nM) compared to *Tc*PARP (25 nM) [17] and *h*PARP-1 (1 nM) can be explained by the amino acid difference as a conserved alanine is replaced by a serine (Ser461) in *Tb*PARP, leading to unfavorable interactions. EB-47 and 4-ANI have been previously reported to inhibit PAR synthesis in human cells [41, 42] and presented 10-fold selectivity for *Tb*PARP over *h*PARP-1. However, despite the displayed *in vitro* enzymatic inhibition they were not able to reduce PAR synthesis in the parasite. Only Olaparib and Rucaparib were able to reduce PAR formation in both procyclic and bloodstream forms. This might be explained by variations in the capability of these drugs to penetrate the parasites external membrane or by other mechanisms that allow metabolic detoxification of these inhibitors by the parasite [17]. Surprisingly, only Rucaparib at the highest concentration (15 μM) rendered a visible impact on the growth rate of procyclic parasites in the conditions tested here. This concentration was in a range similar to that exerted by Nifurtimox, a compound that has been recently incorporated as a combination therapy against Sleeping Sickness [37, 38].

On the other hand, the absence of an active *Tb*PARP in procyclic parasites has no consequences on viability, which has also been proved by our RNAi experiments. This evidence is in agreement with those results reported by an RNAi target sequencing study [43]. The result obtained here differs from the one reported for *T. cruzi* epimastigotes *in culture* where Olaparib at 25 nM concentration led to a 50 % decrease in growth of the parasites [17], indicating different roles of PARP enzymes in these trypanosomatids.

PARP down-regulation did not interfere with normal cell growth but the cytotoxicity induced by H_2_O_2_ was reduced both by PARP down-regulation and by Olaparib. Cell death can be achieved through an apoptotic or a necrotic pathway, which depends on the type of cell and the concentration of the H_2_O_2_ involved. There is evidence that PAR acts as a switch not only between survival pathways but also between cell death signalling, mediating a particular cellular response against a specific DNA damage [2]. This mechanism was described by us for the trypanosomatid *T. cruzi* [34] where PARP carries out a differential function in genomic damage-response. Despite DNA repair mechanisms are not totally clarified in these parasites, many pieces of this puzzle are available in the literature [44, 45]. Here we have demonstrated that *Tb*PARP is activated under a genotoxic stimulus and migrates to the nucleus. This phenomenon of translocation has also been demonstrated in *T. cruzi* by our group [34]. An antagonic role can be assigned to PARP function under stress conditions. When the levels of genomic harm are low, PARP activation plays a protective role. Otherwise, substantial genotoxic stimulus induces loss of viability and cell death. PAR is therefore the molecular switch between different cell death pathways [8]. In this regard, PARP inhibition could prevent the massive reduction of NAD^+^ and thus prevent the energy store depletion [46] that leads to the loss of viability induced by high doses of H_2_O_2_. Our results obtained with Olaparib, PARP- silenced and PARP over-expressing parasites are in line with this reasoning.

The real function of PARG regarding cell death is controversial and some authors have reported that PARG inhibition provides protection to oxidative-stressed neuronal cells [47]. Studies based on PARG-silenced cells show conflicting results ranging from protection to lack of effect using different cytotoxic agents [13, 46]. PARG suppression would lead to PARP inhibition by auto-PARylation. Although we have demonstrated auto-PARylation in *T. cruzi*, this mechanism was not established in *T. brucei* yet. On the other hand, cell death is augmented in PARG-deficient cells which are impaired to repair breaks in one and both strands, pointing out the PARG critical role in DNA damage response [12–14]. Although a function for PARG in regulation of oxidative stress-induced cell death has not been clarified, alteration of PAR cycle would lead to a delay in DNA repair and would explain the increased sensitivity to genotoxic stimuli.

PARP over-expressing and PARG-silenced cells evidenced PAR accumulation in the nucleus, even in absence of oxidative stress. Increased nuclear PAR in parasites with abrogated PARG demonstrates an active polymer synthesis in procyclic parasites and indicates that PARG is the main PAR cycling enzyme. After a cytotoxic stimulus, both transgenic parasites presented a different pattern in the PAR levels. Moreover, these parasites with an altered PAR metabolism demonstrated an increased sensitivity towards oxidative stress, and the changes obtained in the pattern of membrane permeability and PI incorporation indicate that PARP and PARG would mediate apoptosis-like to necrosis death switch. The duality of PAR being a molecular switch between both life and cell death has been proposed by many authors. PAR is involved in DNA damage response. However, an augmented PAR formation could deplete NAD^+^ and consequently remove ATP from the cell, inducing cell death. We showed that not only the quantity of the synthesized PAR might have an effect on parasite survival, but also its particular nuclear localization. Andrabi and colleagues described that PAR could be toxic by itself studying the effect of the presence of *in vitro* synthesized PAR delivered inside the cells with a system based on lipids [48].

Cells die by different mechanisms and this is a subject of many investigations. Among them, “parthanatos” happens when hPARP-1 is over activated; and is mainly related to synthesis of PAR and accumulation, mitochondrial depolarization, AIF translocation to the nucleus and caspases activation; although the latter is not mandatory [49]. Very little is known about the regulation and the type of parasite death in trypanosomatids. Moreover, caspases, calpains and cathepsins, as well as serine proteases and important mediators such as AIF, have not been identified yet; making the characterization of different death pathways even harder.

Summing up, disrupted PAR metabolism with accumulated polymer in the nucleus has deleterious consequences to the parasites when exposed to genotoxic stimulus.

## Conclusions

*Trypanosoma brucei*, like *Trypanosoma cruzi,* has only one enzyme PARP. However, unlike *T. cruzi*, protein inhibition or the lack of PARP in *T. brucei* has no consequences on normal replication. This finding shows substantial dissimilarity regarding PARP role in different trypanosomatids’ cell cycle.

*Tb*PARP activity seems to be very dynamic, even in standard conditions. However, after genotoxic stimulus PAR accumulation in the nucleus is observed. Our results demonstrate that *Tb*PARP down regulation reduced cytotoxicity induced by H_2_O_2_; and, accordingly, *Tb*PARP over expression led to a higher sensitivity against the mentioned agent.

PARG, the main PAR hydrolyzing enzyme, has an important function in maintenance of PAR levels and in regulation of cell death following a genotoxic insult. Our results have shown that in undamaged parasites PARG deficiency does not result lethal; however PAR accumulation in the nucleus conferred an increased sensitivity towards oxidative stress. In all cases, the presence of high levels of nuclear PAR constituted a signal leading to a different cell death pattern. This confirms that the PAR polymer is cytotoxic by itself.

Overall we have demonstrated the importance of PAR metabolism in cell death induced by oxidative stress, although further investigation needs to be done to clarify the mechanisms involved.

## Additional files

Additional file 1: Optimization of assay conditions for TbPARP *in vitro* activity. NAD^+^ consumption was measured by a decrease in fluorescence in a 96-well plate. Triplicates were obtained for every data point, and expressed as means and standard deviations. A) NAD^+^ consumption by *Tb*PARP was measured as a function of pH using a variety of buffering agents as indicated on the x-axis. B) NAD^+^ consumption was determined in 100 mM Na_2_HPO_4_/NaH_2_PO_4_ buffer at pH 7 at different concentrations of activated DNA. C) NAD^+^ consumption by *Tb*PARP (10 nM) was determined in 100 mM Na_2_HPO_4_/NaH_2_PO_4_ buffer at pH 7 supplemented with 25 μg/mL activated DNA in the presence of chloride salts of different divalent cations (2 mM). (TIF 3064 kb) Additional file 2: Table S1: PARP inhibitor like compounds tested. (XLSX 12 kb) Additional file 3: Transgenic RNAi-*Tb*PARP procyclic parasites. A) Representative growth curve shows cell density of tetracycline 3 day-induced (+Tet) and non-induced (− Tet) RNAi-*Tb*PARP procyclic parasites of two different clones, 1 and 2, monitored for 11 and 9 days, respectively. B) Representative growth curve shows cell density of tetracycline 3 day-induced (+Tet) and non-induced (− Tet) RNAi-*Tb*PARP procyclic parasites in presence or in absence of 290 nM Olaparib, monitored for 6 days. C) Northern blot analysis of RNAi-*Tb*PARP procyclic parasites on days 3, 6, 9 and 11 post-induction (p.i.) compared to non-induced cells at identical time points. 30 μg of total RNA was loaded on every lane. Hybridization was performed with a [^32^P]dCTP-labelled *Tb*PARP fragment made by random priming the same PCR product used as insert in the p2T7-177 vector. Ribosomal RNA levels confirmed total RNA equal amounts in each lane. The band corresponding to *Tb*PARP mRNA is shown by an arrow and it is also present in wild type parasites (WT). D) Western blot analysis of 2 × 10^6^ three day-induced (+ Tet) and non-induced (- Tet) RNAi-*Tb*PARP cell equivalents revealed with specific 1:100 anti-*Tb*PARP polyclonal antibody (GeneScript). The membrane stained with Red Ponceau was used as a loading control. (TIF 888 kb) Additional file 4:
*Tb*PARG in *Trypanosoma brucei.* A) *Tb*PARG localization in untreated (control) and in procyclic cultures exposed to 500 μM H_2_O_2_ for 10 min. IFI was carried out as reported in our previous work [33]. *Tb*PARG was identified with our home-made antibody against *Tc*PARG [33]; and PAR was identified with a commercial antibody against PAR (BD). White bar represents 50 μm. B) Western blot analysis of 40 μg protein per lane revealed with a commercial anti-PARG antibody (Antibody Verify) in *T. brucei* procyclic (PC) and bloodstream (BST) forms. The arrow indicates the band with the expected molecular weight (approximately 60 kDa). The membrane stained with Red Ponceau was used as a loading control. (TIF 4272 kb) Additional file 5: Transgenic *Tb*PARP over-expressing and *Tb*PARG down-regulated procyclic parasites. A) Western blot analysis of 3 day-induced (Tet+) and non-induced (Tet-) *Tb*PARP over-expressing (p2216-*Tb*PARP-eYFP) parasites. 2 × 10^6^ cell equivalents were revealed with specific 1:500 mouse monoclonal antibody directed against GFP (Santa Cruz) (Arrow). Staining of the membrane with Red Ponceau was used as a loading control. B) Over-expression of *Tb*PARP-eYFP fusion protein in a 3 day-induced culture (p2216 *Tb*PARP) was also assessed by IFI, detecting eYFP fluorescence. C) Western blot assessment of 3 day-induced (Tet+) and non-induced (Tet-) RNAi-*Tb*PARG (p2T7 *Tb*PARG) parasites. 2 × 10^6^ cell equivalents were revealed with commercial 1:500 rabbit antibody directed against PARG proteins (Antibody Verify) (Arrow). The same membrane revealed with anti-Tubulin antibody was used as a loading control. (TIF 18,172 kb) Additional file 6: Cell survival in eYFP over-expressing (p2216) cultures subjected to hydrogen peroxide (H_2_O_2_) treatment. As a control of the experiment carried out with *Tb*PARP-eYFP over-expressing (p2216-*Tb*PARP) cultures (Fig. 4c), cell survival of 3 day-induced (Tet+) and non-induced (Tet -) eYFP over-expressing (p2216) cultures was studied by measuring parasite motility 6 h after treatment with different hydrogen peroxide concentrations. Statistical significance of three independent experiments was assessed in comparison to the control group (* p < 0.05). (TIF 700 kb) Additional file 7: Nuclear PAR signal quantification in *Tb*PARP-eYFP over-expressing and *Tb*PARG down-regulated procyclic parasites. PAR and DAPI (control) fluorescence was measured following ImageJ instructions. Nuclear area was selected from ten to fifteen parasites and integrated density (IntDen) was calculated. Three different regions per parasite were also selected next to the nuclei as a background. CTCF was obtained as described in Fig. 5b. A and B) *Tb*PARP-eYFP over-expressing parasites. C and D) RNAi-*Tb*PARG parasites. Student’s Test was performed and significance of the nuclear signal in (Tet +) versus (Tet -) parasites is indicated (*** p < 0.001; **, p < 0.01). (TIF 5373 kb)

## Abbreviations

PAR: Poly(ADP-ribose)
PARG: Poly(ADP-ribose) glycohydrolase
PARP: Poly(ADP-ribose)polymerase
*T. brucei*: *Trypanosoma brucei*
*T. cruzi*: *Trypanosoma cruzi*
*Tb*PARG: *Trypanosoma brucei* Poly(ADP-ribose)glycohydrolase
*Tb*PARP: *Trypanosoma brucei* Poly(ADP-ribose)polymerase
*Tc*PARP: *Trypanosoma cruzi* Poly(ADP-ribose)polymerase
