# Supplementary figures and images for: Disrupted ADP-ribose metabolism with nuclear Poly (ADP-ribose) accumulation leads to different cell death pathways in presence of hydrogen peroxide in procyclic Trypanosoma brucei
Source: Parasit Vectors. 2016 Mar 23;9:173. doi: 10.1186/s13071-016-1461-1 (PMC4806436; doi:10.1186/s13071-016-1461-1)

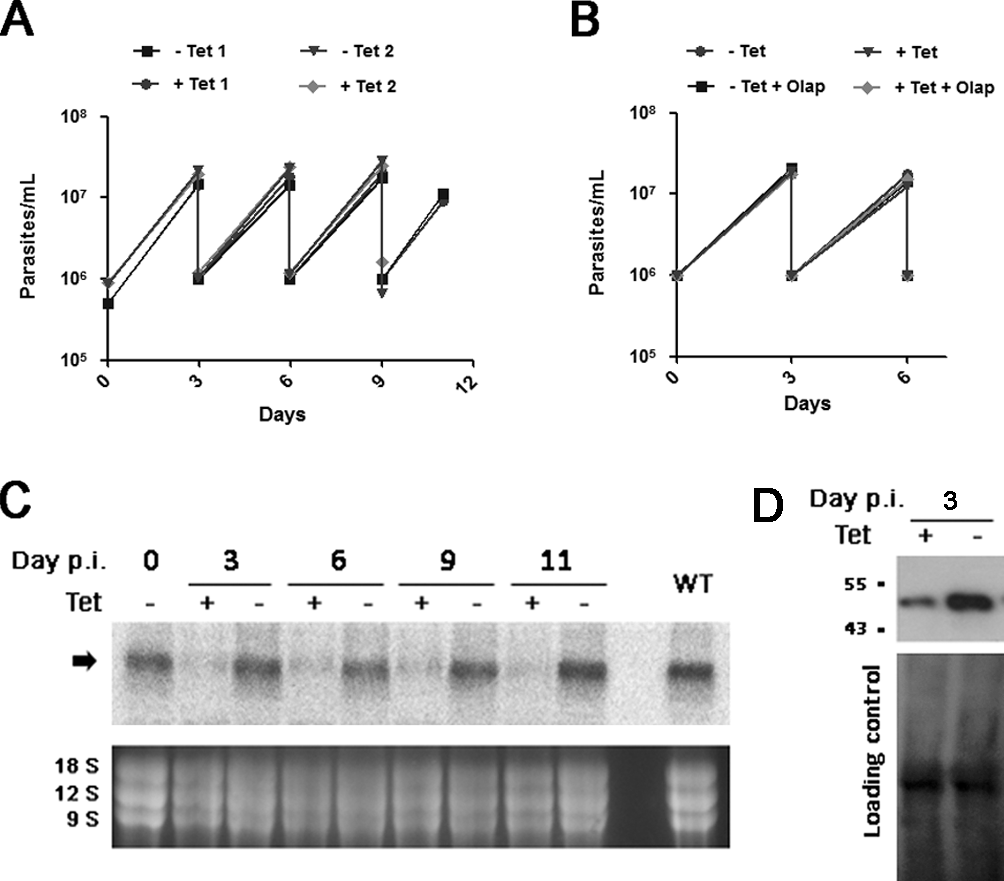

Supplement: Additional file 3: — Transgenic RNAi-TbPARP procyclic parasites. A) Representative growth curve shows cell density of tetracycline 3 day-induced (+Tet) and non-induced (− Tet) RNAi-TbPARP procyclic parasites of two different clones, 1 and 2, monitored for 11 and 9 days, respectively. B) Representative growth curve shows cell density of tetracycline 3 day-induced (+Tet) and non-induced (− Tet) RNAi-TbPARP procyclic parasites in presence or in absence of 290 nM Olaparib, monitored for 6 days. C) Northern blot analysis of RNAi-TbPARP procyclic parasites on days 3, 6, 9 and 11 post-induction (p.i.) compared to non-induced cells at identical time points. 30 μg of total RNA was loaded on every lane. Hybridization was performed with a [32P]dCTP-labelled TbPARP fragment made by random priming the same PCR product used as insert in the p2T7-177 vector. Ribosomal RNA levels confirmed total RNA equal amounts in each lane. The band corresponding to TbPARP mRNA is shown by an arrow and it is also present in wild type parasites (WT). D) Western blot analysis of 2 × 10^6^ three day-induced (+ Tet) and non-induced (- Tet) RNAi-TbPARP cell equivalents revealed with specific 1:100 anti-TbPARP polyclonal antibody (GeneScript). The membrane stained with Red Ponceau was used as a loading control. (TIF 888 kb) [file 13071_2016_1461_MOESM3_ESM.tif]

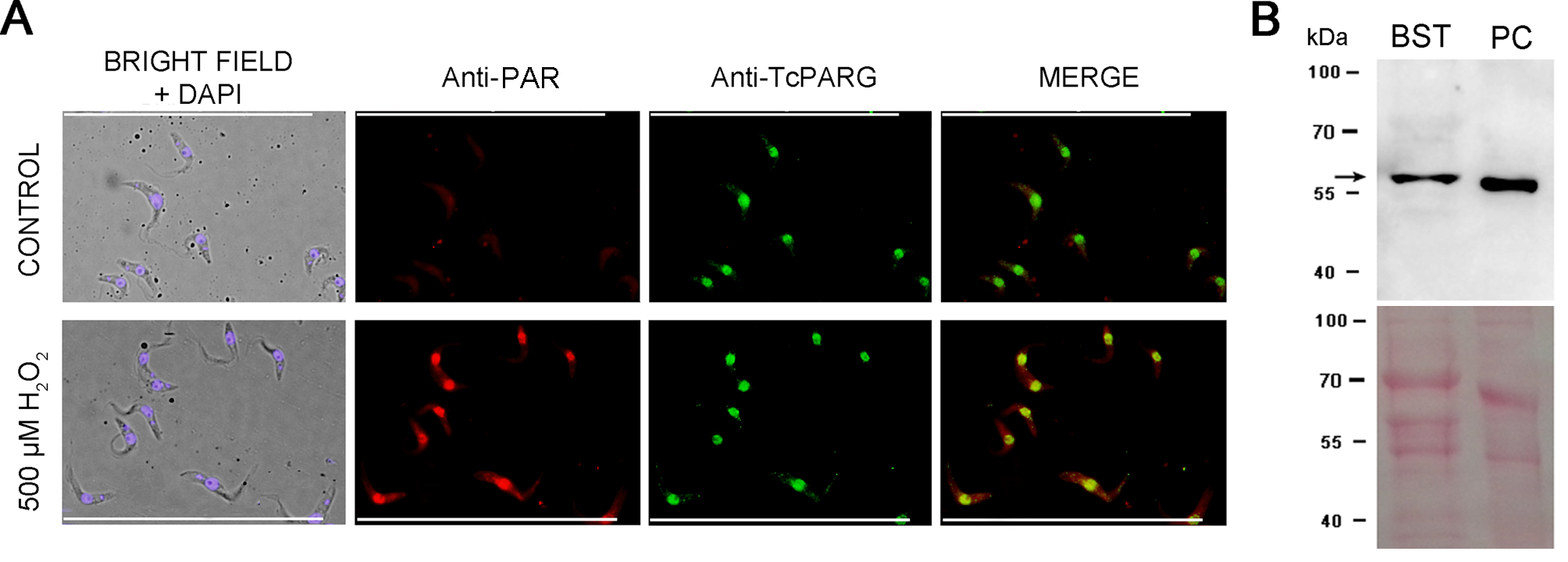

Supplement: Additional file 4: — TbPARG in Trypanosoma brucei. A) TbPARG localization in untreated (control) and in procyclic cultures exposed to 500 μM H2O2 for 10 min. IFI was carried out as reported in our previous work [33]. TbPARG was identified with our home-made antibody against TcPARG [33]; and PAR was identified with a commercial antibody against PAR (BD). White bar represents 50 μm. B) Western blot analysis of 40 μg protein per lane revealed with a commercial anti-PARG antibody (Antibody Verify) in T. brucei procyclic (PC) and bloodstream (BST) forms. The arrow indicates the band with the expected molecular weight (approximately 60 kDa). The membrane stained with Red Ponceau was used as a loading control. (TIF 4272 kb) [file 13071_2016_1461_MOESM4_ESM.tif]

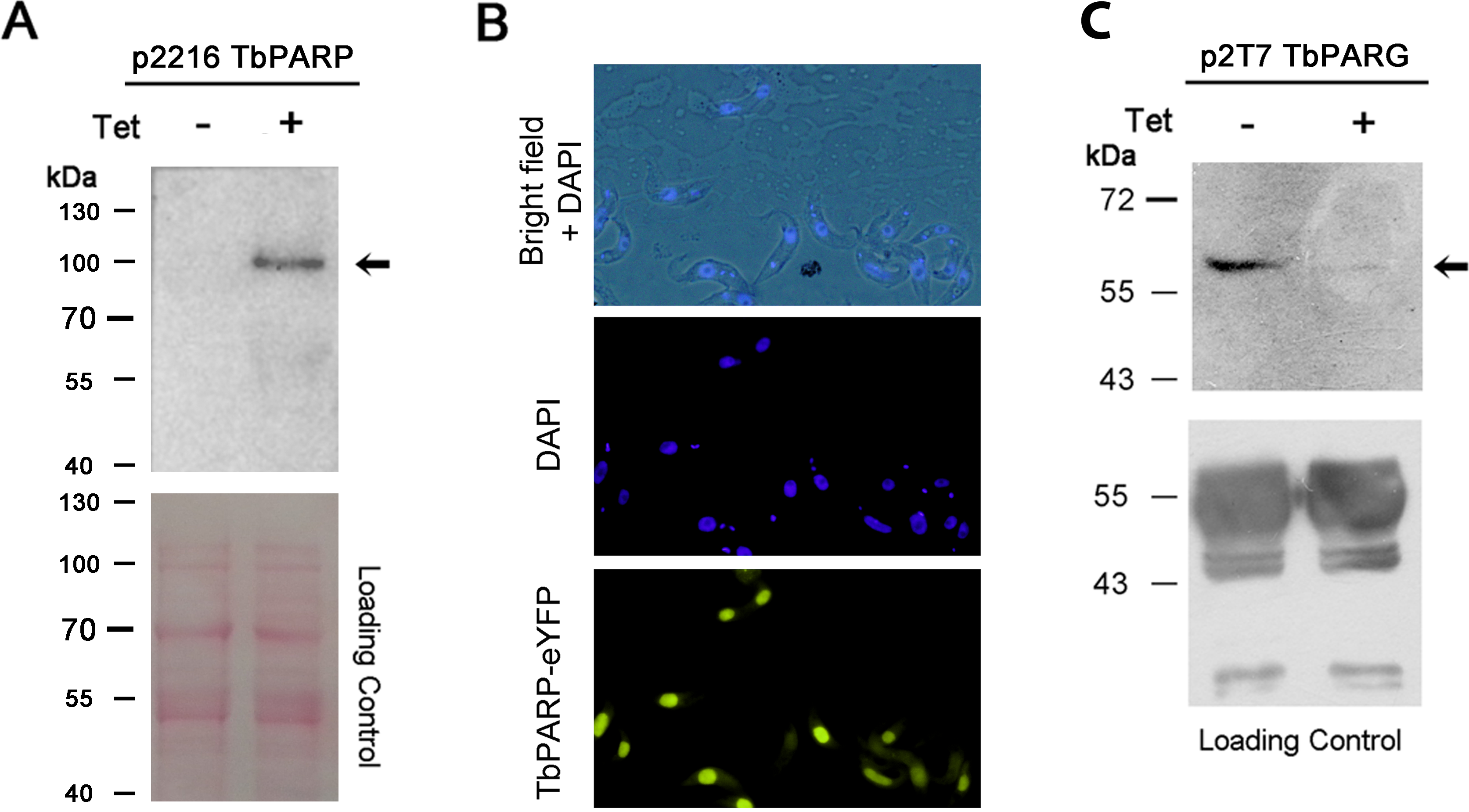

Supplement: Additional file 5: — Transgenic TbPARP over-expressing and TbPARG down-regulated procyclic parasites. A) Western blot analysis of 3 day-induced (Tet+) and non-induced (Tet-) TbPARP over-expressing (p2216-TbPARP-eYFP) parasites. 2 × 10^6^ cell equivalents were revealed with specific 1:500 mouse monoclonal antibody directed against GFP (Santa Cruz) (Arrow). Staining of the membrane with Red Ponceau was used as a loading control. B) Over-expression of TbPARP-eYFP fusion protein in a 3 day-induced culture (p2216 TbPARP) was also assessed by IFI, detecting eYFP fluorescence. C) Western blot assessment of 3 day-induced (Tet+) and non-induced (Tet-) RNAi-TbPARG (p2T7 TbPARG) parasites. 2 × 10^6^ cell equivalents were revealed with commercial 1:500 rabbit antibody directed against PARG proteins (Antibody Verify) (Arrow). The same membrane revealed with anti-Tubulin antibody was used as a loading control. (TIF 18,172 kb) [file 13071_2016_1461_MOESM5_ESM.tif]

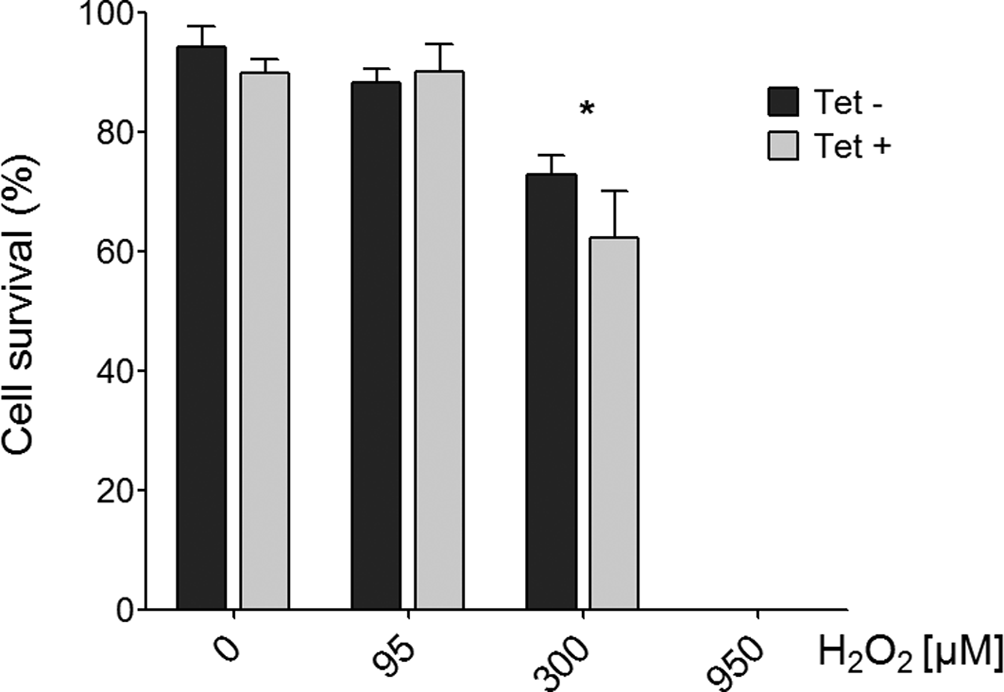

Supplement: Additional file 6: — Cell survival in eYFP over-expressing (p2216) cultures subjected to hydrogen peroxide (H2O2) treatment. As a control of the experiment carried out with TbPARP-eYFP over-expressing (p2216-TbPARP) cultures (Fig. 4c), cell survival of 3 day-induced (Tet+) and non-induced (Tet -) eYFP over-expressing (p2216) cultures was studied by measuring parasite motility 6 h after treatment with different hydrogen peroxide concentrations. Statistical significance of three independent experiments was assessed in comparison to the control group (* p < 0.05). (TIF 700 kb) [file 13071_2016_1461_MOESM6_ESM.tif]

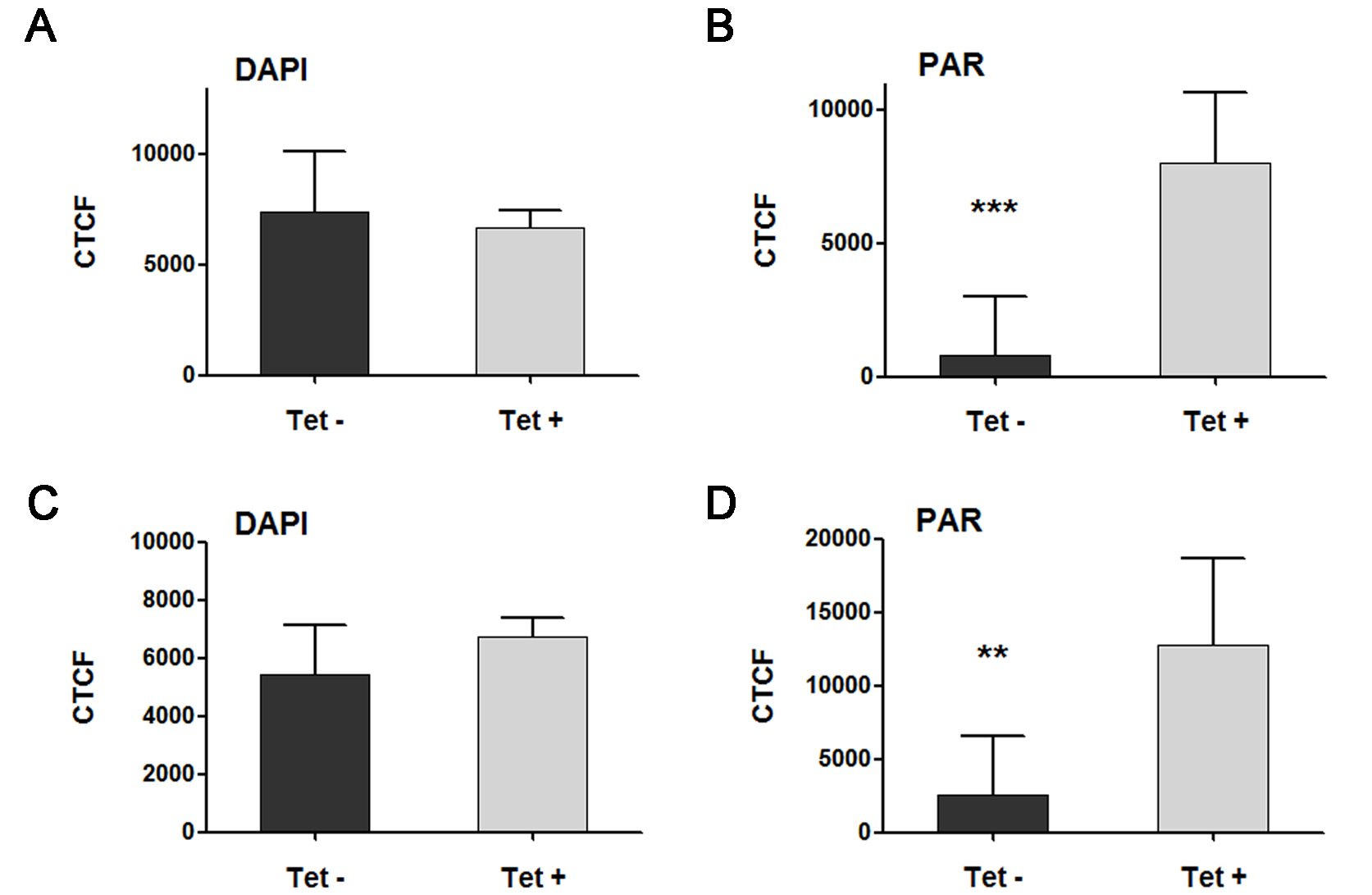

Supplement: Additional file 7: — Nuclear PAR signal quantification in TbPARP-eYFP over-expressing and TbPARG down-regulated procyclic parasites. PAR and DAPI (control) fluorescence was measured following ImageJ instructions. Nuclear area was selected from ten to fifteen parasites and integrated density (IntDen) was calculated. Three different regions per parasite were also selected next to the nuclei as a background. CTCF was obtained as described in Fig. 5b. A and B) TbPARP-eYFP over-expressing parasites. C and D) RNAi-TbPARG parasites. Student’s Test was performed and significance of the nuclear signal in (Tet +) versus (Tet -) parasites is indicated (*** p < 0.001; **, p < 0.01). (TIF 5373 kb) [file 13071_2016_1461_MOESM7_ESM.tif]
